# Community-based biological control of malaria mosquitoes using *Bacillus thuringiensis* var. *israelensis* (Bti) in Rwanda: community awareness, acceptance and participation

**DOI:** 10.1186/s12936-017-2046-y

**Published:** 2017-10-03

**Authors:** Chantal Marie Ingabire, Emmanuel Hakizimana, Alexis Rulisa, Fredrick Kateera, Bart Van Den Borne, Claude Mambo Muvunyi, Leon Mutesa, Michelle Van Vugt, Constantianus J. M. Koenraadt, Willem Takken, Jane Alaii

**Affiliations:** 1 Department of Health Promotion, Maastricht University, Maastricht, The Netherlands; 2 Medical Research Center, Rwanda Biomedical Center, Kigali, Rwanda; 3 Malaria & Other Parasitic Diseases Division, Rwanda Biomedical Center, Kigali, Rwanda; 4 Laboratory of Entomology, Wageningen University, Wageningen, The Netherlands; 5 Department of Cultural Anthropology and Development Studies, Radboud University Nijmegen, Nijmegen, The Netherlands; 6 Academic Medical Center, Amsterdam, The Netherlands; 7 College of Medicine and Health Sciences, University of Rwanda, Kigali, Rwanda; 8 Context Factor Solutions, Nairobi, Kenya

**Keywords:** Malaria, Community knowledge, Acceptance, Participation, Larval source management, *Bacillus thuringiensis israelensis*, Rwanda

## Abstract

**Background:**

Targeting the aquatic stages of malaria vectors via larval source management (LSM) in collaboration with local communities could accelerate progress towards malaria elimination when deployed in addition to existing vector control strategies. However, the precise role that communities can assume in implementing such an intervention has not been fully investigated. This study investigated community awareness, acceptance and participation in a study that incorporated the socio-economic and entomological impact of LSM using *Bacillus thuringiensis* var. *israelensis* (Bti) in eastern Rwanda, and identified challenges and recommendations for future scale-up.

**Methods:**

The implementation of the community-based LSM intervention took place in Ruhuha, Rwanda, from February to July 2015. The intervention included three arms: control, community-based (CB) and project-supervised (PS). Mixed methods were used to collect baseline and endline socio-economic data in January and October 2015.

**Results:**

A high perceived safety and effectiveness of Bti was reported at the start of the intervention. Being aware of malaria symptoms and perceiving Bti as safe on other living organisms increased the likelihood of community participation through investment of labour time for Bti application. On the other hand, the likelihood for community participation was lower if respondents: (1) perceived rice farming as very profitable; (2) provided more money to the cooperative as a capital; and, (3) were already involved in rice farming for more than 6 years. After 6 months of implementation, an increase in knowledge and skills regarding Bti application was reported. The community perceived a reduction in mosquito density and nuisance biting on treated arms. Main operational, seasonal and geographical challenges included manual application of Bti, long working hours, and need for transportation for reaching the fields. Recommendations were made for future scale-up, including addressing above-mentioned concerns and government adoption of LSM as part of its vector control strategies.

**Conclusions:**

Community awareness and support for LSM increased following Bti application. A high effectiveness of Bti in terms of reduction of mosquito abundance and nuisance biting was perceived. The study confirmed the feasibility of community-based LSM interventions and served as evidence for future scale-up of Bti application and adoption into Rwandan malaria vector control strategies.

## Background

Indoor residual spraying (IRS), use of long-lasting insecticidal-treated nets (LLINs) as well as prompt and correct use of artemisinin-based combination therapy (ACT) are methods widely deployed for malaria control in Rwanda, similar to many other settings in Africa [1]. However, these control strategies are becoming less effective with increasing resistance to the most commonly used insecticides and drugs [2]. Targeting indoor transmission alone will not bring malaria to elimination especially in settings where a trend towards outdoor biting by mosquito vectors is observed [3]. A package of integrated malaria vector control interventions targeting the different stages of mosquitoes, including outdoor- and indoor-biting vectors, is desired [4, 5]. This implies that there is a need to deploy additional tools to interrupt the vector life cycle, preferably at the larval stage, before dispersion to human habitations [2, 5, 6]. Larval source management (LSM), using chemical and biological substances, has been recommended by WHO, and is being used to supplement malaria elimination efforts along with IRS and LLINs [7]. These methods have been shown to be viable and cost effective in some African settings, particularly in situations where larval habitats are defined and accessible by hand application [5, 8–12]. One of the biological agents that has been successfully used to target the larval stage of mosquitoes is the bacterial pathogen *Bacillus thuringiensis israelensis* (Bti) [13, 14]. Bti has a low potential for resistance when compared to chemical larvicides and is safe for human health [15–19]. Its larvicidal action resides in its highly specific toxicity to *Anopheles*, *Culex* and *Aedes* larvae, which occurs via ingestion of activated toxic proteins produced by Bti. Mosquito larvae die as a result of the destruction of cell membranes lining the insect midgut [16, 19].

Most of the previous interventions in LSM employed vertical management with only a few using a community-based approach [20, 21]. To date, no study has been undertaken to examine the effectiveness of LSM through larviciding with Bti on malaria control in Rwanda. In the framework of a community-based malaria elimination project implemented in 2012 [22], a comparative community-based LSM intervention using Bti was conducted in Ruhuha, Rwanda, in 2015. Recognizing that working with communities, not only as groups directly affected by malaria but also as critical partners for creating and maintaining larval habitats, would lead to a more effective programme [23], the intervention deployed a community-based approach with in-built baseline and endline socio-economic study for outcome evaluation. The current paper describes baseline and endline community awareness and acceptance of the LSM intervention using Bti. Secondly, the paper highlights challenges faced during the implementation and formulates recommendations for future community-based larviciding activities on a larger scale. A companion paper (Hakizimana et al.) will separately describe the entomological impact of this community-based biological control programme.

## Methods

### LSM using Bti

**Fig. 1:**
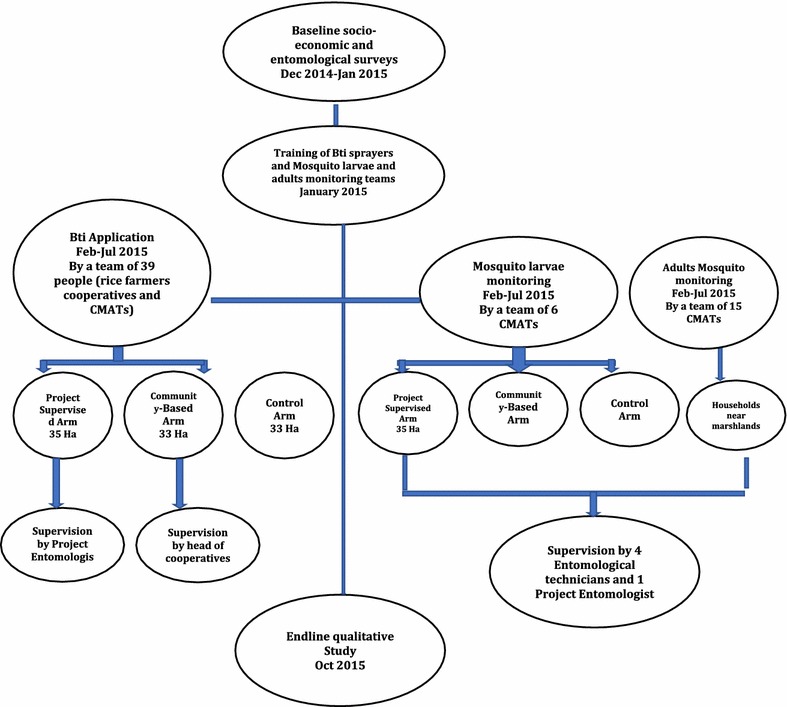
Bti Intervention design and implementation

A community-based LSM using water-dispersible granules of Bti (Vectobac^®^) was established and deployed weekly for a duration of 6 months from February to July 2015 (equivalent to one rice growing season). The aim was to interrupt the development of malaria vector populations by targeting the aquatic larval stages. The intervention was deployed in four local marshlands (mainly occupied by rice fields) and 19 peridomestic water dams. To allow for comparative measurements, three study arms were considered: one marshland of 33 ha with no LSM activities (control) and four marshlands divided equally over two intervention arms (with LSM activities). The first intervention arm with a total area of 35 ha was directly implemented and supervised by the project research team (project supervised or PS). In the second intervention arm of 33 ha, the intervention was directly organized and supervised by community members themselves (community-based: CB). Inclusion criteria for spraying team members included being a rice farmer and a member of a local community malaria action teams (CMATs). CMATs were previously initiated at village level to identify local malaria-related problems and participate in identification of possible solutions. The teams comprised the village leader, a community health worker and youth representative. In total, 39 sprayers were selected for both intervention arms. For monitoring and evaluation purposes, larval and adult mosquitoes were collected on a weekly basis by 21 other members of CMATs closely supervised by four, trained, entomology technicians and one project entomologist (Fig. 1). Prior to implementation, training sessions were held for both intervention arms as well as larval and adult mosquito monitoring teams. Training covered topics on malaria epidemiology, biology of the vector, actual larviciding protocols, as well as monitoring and evaluation.

### Study site

Ruhuha sector is situated in Bugesera District, in the Eastern Province of Rwanda. The population is 23,893 individuals living in 5098 households. The area is a moderate malaria-endemic zone with prevalence estimated at 23% symptomatic and 5% asymptomatic cases at health centre and household level, respectively [24, 25]. The primary malaria vectors in the area are *Anopheles arabiensis* and *Anopheles gambiae* s.s. [25]. The sector has five marshland areas (Nyagafunzo, Nyaburiba, Kibaza, Gatare, Kizanye) in which agriculture (mainly rice farming) is predominantly practised. Common water bodies found in the area are rice paddies, temporary wetlands, ditches, and water streams.

### Study procedures

#### Stakeholder pre-engagement meetings

Community engagement activities were conducted at three different levels with the objective to inform and validate the Bti intervention protocol and create awareness among the local population. The first meeting was held in December 2014 with the Ruhuha sector administrative and health authorities. Subsequently, two informative sessions with existing project-related CMATs as well as rice farmers’ cooperative members were conducted. During the sessions, a summary of the intervention protocol was provided, the role of every member was highlighted and clarifications provided accordingly.

#### Quantitative study: socio-economic baseline questionnaire

A previous stakeholder analysis by the project team identified four rice farmers’ cooperatives with 1914 members from which a cluster random sample of rice farmers for the socio-economic baseline survey was drawn, and conducted in January 2015 [26]. With a confidence level of 95% and a risk of error of 5%, a minimum sample size of 320 rice farmers from the four cooperatives and representing the marshlands in which the three intervention arms were to be experimented (control, community-based and project supervised) was calculated for statistical analyses.

A standard structured questionnaire was developed in the local language (*Kinyarwanda*) and pre-tested. A final version of the questionnaire was designed in an electronic form using Open Data Kit [2]. A team of 12 data collectors was trained for 4 days in January 2015 and conducted the fieldwork in close collaboration with the investigators. The interviews were held at each of the four cooperatives’ office. The measurements included demographics, households’ characteristics, general knowledge on malaria transmission, symptoms and prevention, awareness of larvicides, community perceptions towards safety and effectiveness of larvicides, as well as willingness to spend more time in larvicide-related activities in the area.

#### Qualitative study: focus group discussions

To ensure maximum variation in the responses, non-probability sampling was used and an a priori sample target of 5 and 10 focus group discussions (FGD) with 8–10 participants each was selected for baseline and endline studies. The baseline qualitative study was conducted in January 2015, a few days after the quantitative survey was completed, while the endline study was conducted 3 months after the completion of Bti spraying activities (October 2015). Invitation letters were sent to the leaders of groups to select people (males and females) from each rice farmers’ cooperative (taking into consideration the three intervention arms), administrative sector (local leaders), health professionals, Bti sprayers (CB and PS separately), larval and adult mosquito monitoring teams, community health workers (CHWs), CMATs, and lay community. Two trained project team members led the FGDs. Using almost similar topic lists for both baseline and endline studies, data were collected on community knowledge of mosquito reproduction, including the role of people in the creation of breeding sites and on community acceptance of Bti, including potential advantages/disadvantages. Additional questions at endline explored challenges observed while implementing the intervention, differences and similarities during Bti implementation across interventions arms (PS and CB), suggested community participation activities, as well as recommendations for future scale-up of Bti application.

### Ethical considerations

Interviews and discussions were held in *Kinyarwanda* and each interview was conducted after obtaining written informed consent from participants. The larger Malaria Elimination Project, Ruhuha (MEPR) received approvals from the Rwanda National Ethics Committee (385/RNEC/2012) and National Health Research Committee (NHRC/2012/PROT/0015).

### Data analysis

Descriptive analyses from the quantitative data were performed using SPSS version 21.0 (IBM Corp, Armonk, NY, USA). Variables related to demographics, household characteristics, knowledge on malaria transmission, symptoms and prevention, frequency of mosquito bites in marshlands, citing rice fields as a common mosquito-breeding site, having ever heard about larviciding, and perceptions towards safety and effectiveness of larviciding were included into bivariate analyses to determine factors associated with community willingness to spend labour time for larviciding activities (0 = not willing and 1 = willing to contribute an hour or more per day). Significant variables at a screening p value of < 0.25 were included in a final multivariate logistic regression using backward stepwise (likelihood ratio). The variables in the final model were gender, marital status, family size, level of income, time involved in rice farming, capital, perception of rice farming as profitable, knowledge of malaria symptoms ever heard about, LSM and safety of LSM on living organisms.

All data from the FGDs were recorded and transcribed verbatim and translated into English. The translated narratives were coded using QSR Nvivo10 software (QSR International Pty Ltd). Data were analysed mainly from a deductive approach but allowed for additional emerging themes. Selected verbatim quotes were manually checked for accuracy.

## Results

### Quantitative study: socio-economic baseline questionnaire

#### Socio-demographic characteristics

A total of 320 rice farmers with a mean age of 44.4 years participated in the quantitative survey. Males accounted for 54.7% of the participants. Sixty-nine percent of respondents attended primary school and the mean number of household members was 5.7. Seventy-six percent reported rice farming as the most important source of income.

#### Experience with and knowledge about malaria

Of the total sample of 320 participants, 221 (69.1%) reported a fever/malaria experience for one or more household’s members in the 12 months prior to the study. Almost all participants were widely knowledgeable of the correct cause of malaria and cited mosquitoes as the vector of malaria without citing any form of misconceptions (91.9%). Two-hundred and twenty-five (70.3%) were aware of three or more malaria symptoms with the majority citing fever (92.2%). Two-hundred and thirty-two participants (72.4%) were able to identify at least two types of mosquito-breeding sites and 196 (61.3%) mentioned at least three effective ways of preventing malaria.

#### Awareness and perceptions about LSM

Two-hundred ninety-seven farmers (92.8%) reported rice fields as a common mosquito-breeding site in the area and 294 (91.9%) experienced frequent mosquito bites while in or around rice fields. Only 41 (13%) of the participants were aware of LSM using biological larvicides before it was introduced in the area. However, due to the pre-engagement sessions in which the programme was introduced to the rice farmers prior its implementation, 288 (90%), 284 (88.8%) and 268 (83.8%) reported a high level of perceived safety of Bti to rice consumers, rice farmers as well as other living organisms, respectively. Similarly, 308 (96.2%) and 311 (97.2%) felt confident of the efficacy of larvicides in regard to the reduction of mosquito and malaria transmission, respectively.

#### Rice farmers’ willingness to physically participate in Bti application

Two-hundred thirty participants (72.5%) were willing to make time (one hour or more per day) during future Bti application. A multivariate regression analysis demonstrated that those with knowledge of four malaria symptoms (OR = 3.115, p < 0.001), perceiving Bti as safe to other living organisms (OR = 2.357, p < 0.025), involved in rice farming for fewer than 15 years (0–5 years, 6–14 years) (OR = 4.939, p < 0.008; (OR = 1.900, p < 0.048), with a lower capital contribution to the cooperative (between 0 and 3000 or 3001 and 20,000 RWF) (OR = 6.103, p < 0.000; (OR = 1.870, p < 0.063 (borderline), and perceiving rice farming as less profitable (OR = 1.843, p < 0.043), were more likely to contribute time for future Bti application (Table 1).

**Table 1:** Univariate and multivariate predictors of rice farmer’s acceptance to contribute extra labour time for future Bti activities

Variable	Univariate OR (95% CI)	*p* value	Multivariate OR (95% CI)	*p* value
Age (years)				
20–34	1			
35–50	1.173 (0.621–2.214)	0.623		
51 +	0.741 (0.381–1.442)	0.377		
Gender				
Male	1.567 (0.957–2.567)	*0.074*		
Female	1			
Marital status				
Married or living together	2.059 (1.094–3.875)	*0.025*		
Single/divorced/widow	1			
Educational level				
None	1			
Primary	1.607 (0.921–2.806)	0.095		
Post-primary/vocational	1.059 (0.182–6.156)	0.949		
Secondary school or higher	1.324 (0.379–4.619)	0.660		
Family size				
1–5 people	1.601 (0.977–2.625)	*0.062*		
6 +	1			
Involvement in rice farming (years)				
0–5	6.344 (2.061–19.529)	*0.001*	4.939 (1.504–16.218)	*0.008*
6–14	3.229 (1.889–5.521)	0.000	1.900 (1.007–3.587)	*0.048*
15 +	1			
Household income from rice cultivation				
0–50%	2.155 (1.226–3.788)	*0.008*		
51% +	1			
Capital given for cooperative membership				
0–3000 RWF	7.765 (3.697–16.308)	*0.000*	6.103 (2.639–14.113)	*0.000*
3001–20,000 RWF	2.144 (1.211–3.794)	*0.009*	1.870 (0.966–3.620)	*0.063*
20,001 RWF+	1			
Rice profitable				
No/hardly/modestly	1.995 (1.197–3.325)	*0.008*	1.843 (1.021–3.328)	*0.043*
Yes, very much	1			
Malaria transmission				
No	1			
Mosquito/*Anopheles*	1.441 (0.617–3.365)	0.399		
Malaria symptoms				
1–3 symptoms	1			
4 +	3.114 (1.658–5.848)	*0.000*	3.115 (1.565–6.203)	*0.001*
Frequent mosquito bites in marshlands				
Almost never/once in a while	1			
Often/very often	1.624 (0.572–4.611)	0.362		
Rice cultivation as potential mosquito breeding site				
Not at all important/minor importance	1			
Important/very important	0.784 (0.211–2.916)	0.716		
Ever heard about larviciding				
No	1			
Yes	3.985 (1.377–11.534)	*0.011*		
Larviciding safety on rice consumers				
None/little	1			
Much/very much	1.557 (0.852–2.846)	0.150		
Larviciding safety on rice farmers				
None/little	1			
Much/very much	1.490 (0.814–2.727)	0.196		
Larviciding safety on living organisms				
None/little	1			
Much/very much	2.442 (1.217–4.901)	*0.012*	2.357 (1.114–4.985)	*0.025*
Larviciding reduce mosquito abundance				
None/little	1			
Much/very much	1.386 (0.827–2.322)	0.215		
Larviciding reduce malaria risk				
None/little	1			
Much/very much	1.003 (0.612–1.644)	0.990		

The italics values in the “univariate” column represent the significant variables at a screening *p* value of < 0.25 that were included in the final multivariate analysis

The italics values in “multivariate” column represent the statistically significant variables in the final model

### Qualitative study: baseline and endline outcomes from FGDs

#### Demographical characteristics

A total of 45 participants (64% male) and 92 (62% male) attended baseline and endline sessions, respectively. Twenty of them attended both baseline and endline. The majority of participants in both studies attended primary school, with 71 and 72% for baseline and endline, respectively.

#### Baseline outcomes: knowledge on mosquito larval habitat

Prior to the Bti application, participants were by-and-large knowledgeable on mosquito larval habitats. Participants commonly reported that mosquito larvae breed in water bodies, particularly stagnant water, such as swamps, where rice is being grown, as well as water- collecting instruments left uncovered in some households.

*“Many times we see mosquito larvae in pots and jerry*-*cans used to collect rain water in our homes. These can be breeding sites for mosquitoes, more especially when left uncovered for long.”*

Lay community FGD, Female, 34 years

The role of people in creating mosquito-breeding sites was acknowledged, such as the existence of water dams in the neighbourhood that are used for crop irrigation, animal drinking places and other water storage and sources for domestic purposes. Community-initiated activities aimed at reducing mosquito abundance, such as clearing breeding places especially in the peri-domestic area, were mentioned. However, participants noted that water dams are an essential part of their livelihood and the best action would be to find ways to minimize breeding therein.

*“We cannot remove dams, because they are very useful in providing water for irrigation, and this has been our campaign to encourage people to practise irrigation of crops so as to fight shortage of foods during the dry season. So we shall be contradicting ourselves telling them to close those dams. Instead I think there should be other measures for killing mosquito larvae in these dams.”*

Local authorities FGD, Male, 38 years

#### Baseline outcomes: perceptions on Bti intervention

Similar to the quantitative study, participants in the baseline study were largely unaware of Bti as part of an integrated vector control (IVM) strategy. However, participants expressed enthusiasm for the intervention following a short description of how Bti works and assurance of its safety to humans and animals. Participants perceived Bti as important when used in addition to IRS and LLINs by reducing the number of mosquitoes that transmit malaria.

*“This is something everyone may be happy with, because if you observe well you will find that the existing methods used were not showing a satisfactory outcome. I feel that attacking mosquitoes at the breeding site will provide better results. Though even other existing methods will continue to be used; but if the added method of destroying mosquito larvae is implemented, a more satisfactory outcome will be realized.”*

Sprayers FGD, Male, 41 years

#### Baseline outcomes: perceived advantages and concerns associated with Bti

Numerous perceived advantages and benefits of Bti were cited, not only for rice farmers, but also for communities as a whole. Participants in the baseline study mentioned that the reduction in number of mosquitoes and malaria will contribute to the relief of community members and the national malaria control programme who could re-allocate time and money for other developmental activities and/or health priorities.

*“Once malaria is reduced and people’s health improves, even economic development will be realized. When people are suffering from malaria, they don’t work, and hence no development. Once malaria is gone for good, there is nothing so good like living in a malaria free world!”*

Local community FGD, Female, 34 years

As opposed to the quantitative survey, some concerns with regard to the safety of Bti for human and rice crops were highlighted in FGDs. Some participants reported that Bti would not be widely adopted by rice farmers if it became apparent that Bti would inevitably kill some small insects living in rice fields called *Inshuti y’umuhinzi*, literally meaning ‘friends of a farmer’, helping to fix fertilizers in the soil (*Rhizobium* bacteria). Furthermore, participants highlighted a possible interaction between Bti, fertilizers and other chemicals to kill pests that attack rice crops, thus possibly resulting in reduced effectiveness of the intervention.

Overall, participants expressed interest in Bti and were eager for ongoing educational activities and willing to provide their contribution to sustain Bti. Participants commonly agreed that the best option would be to contribute equal amounts of money among cooperative members in contrast to a progressive contribution depending on land size. Furthermore, the contribution of free time to work for Bti was found possible but with limitations as community members also have their usual activities.

#### Baseline outcomes: recommendations for Bti implementation

A section of participants provided suggestions for implementation during the intervention-planning phase and for the sustainability of Bti if proven successful. Involvement of the local community was considered a key element for success, as well as training enough teams to ensure full coverage. Involvement of local authorities was deemed essential with the goal of increasing community acceptance and uptake of the intervention. Lastly, embedding Bti activities into rice farming cooperatives in the same way as has been done for chemicals and fertilizers used by rice farmers was deemed critical to its sustainability.

*“If Bti will be applied in rice fields and the people involved in spraying would be outsiders, then it may not work well. I would suggest that people to be involved in the spraying exercise are rice farmers, since they know well how to walk in their fields. Each cooperative should select their people who will spray in their rice fields.”*

 Rice farmers FGD, Male, 40 years

#### Endline outcomes: awareness of Bti

Awareness of Bti has widely increased when compared to the baseline. Participants who were directly involved in the implementation further provided their observations on how Bti operates once sprayed into their marshlands.

*“We were thoroughly educated about mosquito reproduction. We even participated in catching these larvae in swamps in areas where they can be commonly found. We later participated in Bti spraying exercise. What I have observed is that really this intervention works well, because wherever we sprayed, we could go back and check, only to find that all mosquito larvae were dead. And before they die, they first bulge and then burst. So this method is very effective.”*

Sprayer-CB arm, FGD, Male, 33 years

#### Endline outcomes: perceived benefits

Following the implementation of Bti in marshlands and peri-domestic water dams, almost all participants reported a reduction of mosquito abundance (both adults and larvae) for the intervention area and a subsequent reduction in mosquito bites while in or around marshlands and even in their homesteads. Prior to the intervention, working in marshlands was reported to be associated with mosquito bites, often leading to swollen arms, and a reduction in mosquito larvae would allow farmers to work without any interference from mosquitoes.

A section of participants mentioned that despite the existence of other IVM tools, Bti boosted the reduction of malaria in the area. Long-term perceived benefits included economic gains and specifically re-allocation of funds that were spent on malaria cases as highlighted in the baseline. Participants who were not directly involved in any of the related Bti activities highlighted their doubts prior to the intervention. However, they acknowledged the benefits observed throughout and towards the end of the intervention.

*“Generally the programme was so beneficial, especially in reducing mosquitoes. You could find to many mosquitoes swarming around homes in the evenings. In fact, we had no peace. But now we can sit out in the evening and enjoy fresh air. Even when you forget to close the windows, still you find no mosquitoes in the house. This is a benefit enjoyed by the whole sector; even malaria in the whole sector has reduced.”*

Lay community FGD, Female, 60 years.

*“There is something that I noticed with this programme. Before Bti spraying began, we could find mosquitoes from swamps swarming like bees, especially in rainy seasons (March and April). People and animals in this sector had no peace in that period. But now, the whole community is at peace, no more swarming of mosquitoes and this shows how effectively this program has reduced mosquitoes in this sector.”*

Mosquito larvae monitoring FGD, Male, 36 years

Participants noted differences with regard to the amount of product used and effectiveness of Bti in various marshlands and peri-domestic water dams. Rapid effectiveness was observed in marshlands where water was flowing at normal velocity, such as irrigation between sweet potato fields when compared to rice fields. Implementation of Bti in peri-domestic water dams was also found feasible, as they are clearly demarcated and easily accessed by hand-application.

*“We observed and found that there was much change in swamps where the intervention was done. Mosquitoes greatly reduced, some to the extent of zero mosquito larvae, whereas in the control area, mosquito larvae continued to multiply daily.”*

Sprayer- CB arm, FGD, Male, 35 years

As opposed to the initial intervention plan that envisaged independent working teams for both intervention arms and mosquito monitoring teams, it was evident that communication between teams was frequent as a way of improving their performance and reaching the goal of mosquito reduction in the area. For instance, larval mosquito monitoring teams acknowledged having shared with the spraying teams information related to their areas on abundance of mosquito larvae and those that needed intensive spraying compared to others.

*“We could check and record the plots and the field where we found plenty of mosquito larvae and the name of the owner. Then we could direct sprayers where we found larvae. So our relationship was good. Besides, we are all rice farmers from the same cooperatives, so we could discuss and tell them that the plot for so and so has plenty of mosquitoes, the next spray you should concentrate there. After all our goal was to control mosquitoes that transmit malaria in our sector.”*

Mosquito larvae monitoring team FGD, Male, 33 years

Both intervention arms (PS and CB) received similar training prior to Bti implementation, which may explain the lack of specific differences across the arms. However, a number of additional dams, not previously mapped by the project team were identified and sprayed by the CB intervention arm compared to the PS intervention arm.

#### Endline outcomes: challenges regarding Bti application

Both spraying and mosquito larvae monitoring teams reported common operational, seasonal and geographical challenges. Challenges with regard to the application of Bti in the rice fields were noted as mainly due to a wider area with stagnant water, muddy and slippery grounds hindering full coverage by hand application and negatively impacting effectiveness. Weather changes, such as heavy rains, were reported to hinder or delay planned activities. Other challenges included lack of transportation, such as bicycles to and from fieldwork, and long working hours to ensure the coverage within the allocated time.

Most participants in both spraying arms reported shortcomings in scheduling days for working in rice fields. The initial schedule of 2 days per rice field was not enough and increased to 3 days a week. However, 3 days was still regarded as insufficient according to the workload, suggesting a need for a further increase in number of days or workers per rice field.

Some participants, mainly from the CB arm, stated that as opposed to the training that instructed sprayers to stand on ridges between rice fields and spray 10 m to the left and 10 m to the right while continuing to walk on ridges, the reality was that some fields were too large to cover while standing on ridges and required one to enter into the fields. The latter had implications on the time that had been scheduled to spray each field and the amount of product to be used.

*“The challenge we met in Gatare swamp is that there is one big ridge which is difficult to climb; it had been suggested that, they will bring a spraying machine that would help in spraying Bti from that ridge, but it was never brought. Though we tried to spray from the edges of that ridge, we couldn’t reach far enough; hence eliminating mosquito larvae in that area was never possible.”*

Sprayer- CB arm, FGD Male, 35 years

*“There was also a challenge of thirst and hunger, because most of the time we could leave early in the morning sometimes without taking anything and spend the whole day in rice swamps walking and carrying pumps. By the time you finish to return home, one would be so exhausted and feeling sick.”*

 Sprayer- PS arm FGD, Male, 41 years

*“In the swamp where I worked, we met the challenge of thorny plants in the swamp that were hidden in the water such that whenever one makes a step, he/she could step on thorny plant, which could hurt you. That was before they gave us gum boots, but after boots were provided, that problem ended.”*

Mosquito larvae monitoring FGD, Male, 26 years

#### Endline outcomes: community concerns

As opposed to the baseline, no concerns were raised as a result of Bti application on the safety of rice farmers that work daily in the marshlands, the quantity of the harvest, water from marshlands (used for other domestic purposes), and rice pests that normally live in marshlands. Previous elements of trust also played a role in how the community perceived the intervention. For example, some participants felt that leaders would not allow a harmful programme to be deployed among its citizens. Trust between rice farmers and their cooperative leaders facilitated their acceptance level of the intervention and subsequently had an effect on their confidence on the safety and effectiveness of Bti.

Interestingly, participants noted a general reduction in rice harvest in the sprayed area, but also in neighbouring sectors without intervention, most probably due to weather changes.

*“Our cooperatives leaders, who are also rice farmers, assured us that this substance is safe and since we trust them, we agreed. Even after starting the spraying activities, some of us could go to the fields to check if there was no effect to our rice crops, but we found that the substance had no negative effect on our rice.”*

 Rice farmers’ FGD, not involved in spraying, Female, 37 years

*“We faced the problem of poor rice crop yield, mainly due to change of weather and crop disease that dried rice plants. But this was not related to Bti spray in the rice fields. It is a problem that tends to occur often whenever weather changes and this did not affect our sector only, even other sectors in this region were affected*.”

 Sprayer-PS arm, FGD, Male, 39 years

#### Endline outcomes: recommendations for future scale-up of Bti application

A large number of participants highlighted the need for Bti to be scaled-up in terms of area covered, but also in terms of the duration of the intervention. The intervention period was evaluated as short (6 months of coverage) and limited to the Ruhuha area. Neighbouring sectors were reported as having the same geographical features (marshlands) and agricultural practices, such as rice farming. This was regarded as conducive for mosquito breeding, thereby enabling mosquitoes to recolonize the Ruhuha area and impeding the effectiveness of spraying activities. Subsequently, participants suggested that future Bti application should be extended into neighbouring areas.

Based on activities that were planned daily for the last round of Bti, it became apparent that the number of community members involved in spraying activities was not enough to accomplish the task within the allocated time, in spite of long working hours (6–8 h), which implied the need for additional workforce in future programmes.

*“There were very few persons to cover all swamps. They were so scattered such that covering the whole swamp was not possible. For example, in the swamp where we grow our rice, only a small part that has rice crops was sprayed, the bigger portion of the swamp where rice is not grown was not sprayed, because they were few. We wish you could increase the number of sprayers, so as to cover all swamps.”*

Rice farmers’ FGD, not involved in spraying. Female, 44 years

#### Endline outcomes: community mobilization, education and participation

As Bti was perceived as successful with promising results in terms of mosquito reduction (companion paper by Hakizimana et al.), some of the participants expressed their concerns that the use of existing individual preventive measures, such as LLINs, may reduce among community members and suggested a critical need for on-going community sensitization for the use of LLINs and acceptance of IRS at household level.

*“There is a need to explain to them (community members) that mosquito larval control does not take away other malaria control measures, but supplements them. Spraying Bti in rice swamps does not mean that sleeping under bed nets or IRS should stop. Instead they should all continue, if we are to eradicate malaria.”*

 Larvae monitoring team FGD, Male, 26 years

Most participants were willing to provide labour time for implementation once the product (Bti) is made locally available. Some participants were also willing to provide a financial contribution, but noted that this option should be well explored and ensure that the socio-economic status of the local community is taken into consideration.

In the framework of self-reliance in Rwanda, a number of achievements in health, education and economic sectors were highlighted as a result of community involvement and commitment. Examples included the establishment of community-based health insurance (CBHI), the construction of facilities for promotion of basic educational programmes, as well as the establishment of cooperatives for savings and credits at sector level (SACCO). With this in mind, participants expressed their willingness to partly contribute to future Bti activities in collaboration with the Government and other partners. Despite community willingness, some participants mentioned that a contribution should start with rice farmers’ cooperatives as was initially done for the pilot phase and be extended to the rest of the community. Furthermore, it was suggested that cooperative members should actively participate in the selection of implementation teams and agree on allowances to be provided to ensure proper and regular Bti activities.

*“I think through rice farmers’ cooperatives, people would easily contribute towards a mosquito larval control program, simply because members in these cooperatives have a better understanding of contributing to communal activities. Later on, even other citizens could be involved after having seen the example from the rice farmers’ cooperatives*.”

Lay community FGD, Male, 61 years

*“… Once the community understands the programme and then observes the benefits, contributing towards its (LSM programme) sustainability will not be a problem. …. I feel even with the mosquito larval control programme the community can contribute towards its sustainability. For instance, in the initial stage, they may contribute about 50% and the government supports with the other 50% of the costs, but later they would be able to support it 100*%.”

 Local leaders FGD, Male, 32 years

#### Endline outcomes: role of government

Many participants acknowledged a positive impact from the Bti intervention and highlighted the need for the programme to be adopted by the Government as part of its malaria IVM strategy. Local authorities however, noted that the intervention may be as expensive as other vector control measures, such as IRS, costing around US$4 per household. A community contribution may not be enough to cover the full costs of Bti, and there is a need for the Government and its partners to play an active role in the scale-up. Furthermore, it was suggested that the Government should ensure the capacity building of teams that are involved in Bti application in terms of knowledge, skills and materials needed to perform their tasks. On this, participants advised deploying context-based solutions while applying Bti (hand or machine application). Lastly, cross-border and joint collaboration between Rwanda and neighbouring countries was highlighted in the framework of vector control to increase the effectiveness of LSM at large.

*“I do agree that this programme has been effective in reducing malaria in our sector and once it is continued, and combined with other existing malaria control measures, no doubt malaria can be eradicated. I would suggest that the research team presents the research findings to the Government, so that they may consider it in their programmes for malaria control and plan how to implement it in collaboration with the citizens. Not only in this sector, but in the whole country.”*

Local leaders FGD, Male, 48 years

## Discussion

The study used both qualitative and quantitative methods to explore community awareness and acceptance prior to and after Bti application, as well as actual participation in the implementation. Baseline findings suggested a highly positive perception of Bti intervention despite a relatively low level of awareness, as opposed to the endline findings. This finding concurs with that found in Tanzania [4]. Mboera and others reported that only 17% of the participants in their survey were aware of LSM, while being supportive of its implementation and confident about its safety and effectiveness [4]. This case has been mainly related to the pre-engagement meetings that were held among rice farmers before Bti implementation which largely facilitated the community adoption of the intervention as innovative in targeting mosquito sources, while serving as an additional tool to the existing malaria preventive measures. In addition, the trust attributed to the leaders and their vigilance in allowing programmes that are beneficial to the population, coupled with prior involvement of rice cooperative leaders who played a key role in passing on the message to their fellow rice farmers facilitated acceptability of the intervention as mentioned in baseline qualitative study. Moreover, direct observation of intervention outcomes throughout the implementation (e.g., counting larvae) resulted in increased levels of confidence.

Some of the man-made habitats that contain mosquitoes and have an impact on the level of malaria transmission are important for community livelihoods, as was similarly found in Kenya and Brazil [27–29]. The justification of the use of novel LSM strategies to complement personal protection measures must also consider the purpose of these water bodies, such as rice irrigation and domestic water use [27, 28]. A proper understanding of psychosocial and physical environment to enable successful implementation of the innovation is needed [30].

Rice farmers in this study commonly reported a high level of perceived effectiveness of Bti application in their agricultural fields with regard to the reduction of mosquito density and nuisance biting. Involvement of rice farmers’ cooperatives in Bti implementation was unique in the area and yielded findings that should serve as evidence for future scale-up. Findings are also comparable to previous studies in Sri Lanka that highlighted the benefits of farmer field schools that involve pest and vector management strategies to improve both agricultural practices while minimizing environmental risks to health [31, 32]. The same studies reported an increased knowledge of mosquito ecology and disease epidemiology among rice farmers as well as a reduction in the burden of *Anopheles* mosquitoes and malaria transmission, while preserving the ecosystem [31–33]. This stress the importance of an inter-sectoral collaboration between agricultural and health institutions towards development of environmentally sound strategies [29, 34]. Another study conducted in Tanzania focusing on resilience processes in community-based LSM highlighted the vital role of improved stakeholder partnership for effective malaria control [35]. The success of the intervention in Rwandan settings depended on high ownership by the local communities that enabled access to the rice fields, overcoming the issue of security and privacy previously reported in other settings [20, 36, 37].

Some participants agreed to provide financial contribution through respective cooperatives to be effective and also a contribution through labour was suggested by some rice farmer categories. The fact that the relatively more experienced and wealthier rice farmers were found less willing to contribute their labour time might be related to their unavailability for daily rice farming-related activities, which is mainly done by hired workers who are remunerated on a daily basis.

A number of community concerns before the intervention with regard to the safety of Bti included the impact on rice growth and harvest, effects on rice farmers themselves working in the fields, and on rice pests and the water used for other domestic purposes. However, almost all participants agreed that concerns were no longer present after the intervention. Lack of community concerns after the implementation of Bti in this study is also similar to that reported previously where no undesirable consequences of larvicides on non-target organisms were reported in a study conducted in Malaysia [38].

Some challenges have been reported in terms of difficulties to achieve full coverage in areas where geographical characteristics are not favourable. Besides, operational challenges such as a lack of transportation, shortage of personnel involved in spraying activities and tight schedules to cover the allocated area, were all cited. Findings imply that innovative interventions go along with unplanned activities that require a prompt response. Some of the challenges were resolved during the course of the intervention, however future scale-up should consider the maximization of effectiveness by using alternative spraying mechanisms such as the use of powered dispensers/sprayers in areas where hand application is practically impossible. Even drones and/or other motorized machines could be deployed to overcome such challenges and could improve the quality and efficiency of large-scale Bti application [16, 39].

This study has been successful as a result of community collaboration and participation at grassroots level. Personal interaction with stakeholders in the community, such as local health authorities, CMATs and rice farming cooperatives, from the planning, adoption, implementation, and evaluation stages was key and goes in line with the country’s self-reliance strategy to adopt local solutions to local problems. The interaction facilitated the learning approach, increased knowledge, capacity and self-empowerment, which may inform future LSM implementation [4, 6, 8, 40–42]. It is however unclear from this study whether willingness to make time for Bti activities will be translated into actual commitment. Moreover, the findings from this study are context-based and may not necessarily represent geographical diversities across the country.

## Conclusions

This study confirmed the feasibility of community-based vector control programmes which is congruent with other settings in Tanzania. A wider knowledge of LSM coupled with high positive perceptions towards its effectiveness in reducing mosquito density and nuisance, as well as community willingness to participate in future LSM activities were reported throughout the intervention. Further programmes should consider the reported challenges to achieve high levels of commitment and ownership. Furthermore, studies on financial models cognizant of affordability and epidemiological analysis are recommended for comprehensive impact evaluation and policy guidance.

## Abbreviations

ACT: artemisinin-based combination therapy
Bti: *Bacillus thuringiensis* var. *israelensis*
CHWs: community health workers
CMATs: community malaria action teams
IRS: indoor residual spraying
IVM: integrated vector management
LSM: larval source management
LLIN: long-lasting insecticide-treated nets
WHO: World Health Organization
